# The acceptability and effectiveness of a questionnaire for the identification of risk factors for HIV and hepatitis B and C: An observational study in general practice

**DOI:** 10.1080/13814788.2017.1400529

**Published:** 2017-11-27

**Authors:** Amélie Aïm-Eusébi, Emmanuel Prothon, Catherine Majerholc, Diana Barger, Yazdan Yazdanpanah, Jean-Pierre Aubert

**Affiliations:** ^a^ Département de médecine générale, Sorbonne Paris Cité, Univ. Paris Diderot Paris France; ^b^ Département de médecine générale, Univ. Bordeaux Bordeaux France; ^c^ ISPED, Centre INSERM U1219-Bordeaux Population Health, Univ. Bordeaux Bordeaux France; ^d^ Service de maladies infectieuses et tropicales (SMIT), AP-HP, Hôpital Bichat Paris France; ^e^ EA Recherche clinique Ville-Hôpital, Méthodologies et Société (REMES) Paris France

**Keywords:** General practice, HIV, viral hepatitis, risk factors, screening

## Abstract

**Background:** Many people in Europe remain undiagnosed for human immunodeficiency virus (HIV), hepatitis B virus (HBV), hepatitis C virus (HCV).

**Objectives:** To evaluate acceptability and effectiveness of a questionnaire designed to facilitate identification of risk factors for these viruses.

**Methods:** We performed an observational study, in a prospectively enrolled cohort of patients in Paris (France) seen in 2014. Eighteen GPs administered a questionnaire to the first 50 patients, collecting information about risk factors. GPs were randomized into two groups: A (self-administered questionnaire) and B (GP-administered questionnaire). We used the overall response rate to assess the acceptability of the questionnaire. We used the rate of newly identified risk factors and compared the number of tests performed one year before and immediately after the intervention to assess the effectiveness of the questionnaire.

**Results:** 842 patients were randomized: 349 (41.5%) in group A and 493 (58.5%) in group B. Acceptability was 88.5% (95%CI: 86.3–90.6); 93.1% (95%CI: 90.5–95.8) in-group A and 85.2% (95%CI: 82.1–88.3) in group B (*P* = 0.0004). Prevalence of risk factors was 51.8% (95%CI: 48.2–54.4) and 58.3% were newly identified (95%CI: 52.9–63.7). The number of HIV tests performed during the four weeks after intervention increased by 27% compared to the same period one year before (*P* = 0.22). It increased by 113% (*P* = 0.005) and 135% (*P* = 0.005) for HBV and HCV, respectively.

**Conclusion:** The questionnaire proved acceptable and effective in identifying risk factors for HIV, HBV and HCV in general practice.

KEY MESSAGES

- Many people in Europe remain undiagnosed for HIV, HBV and/or HCV.
- A questionnaire designed to facilitate the identification of risk factors can help GPs to screen those who are most at risk.
- Targeted screening is potentially more efficient compared to routine screening.

## Introduction

Many people remain undiagnosed for human immunodeficiency virus (HIV), hepatitis B virus (HBV) and hepatitis C virus (HCV). In Europe, this represents 15% of people living with HIV [1], 45 to 55% of people living with HBV and 20 to 78% of people living with HCV [2]. Early screening for these viruses and timely linkage to care, improved life expectancy and quality of life for HIV-positive individuals, and prevent onward transmission [3]. Early screening is essential. General practitioners (GPs) play a central role in the early detection HIV, HBV and HCV.

Most international guidelines for HIV, HBV and HCV recommend offering targeted screening for those at highest risk, in particular, migrants from endemic countries, men who have sex with men (MSM), people with multiple sexual partners and injected drug users [4–9]. Joint screening for the three viruses is recommended in France [8,9].

Targeted rather than routine testing for HIV, HBV and HCV implies that GPs must determine whether their patients are at risk. Prior research has shown that GPs often fail to routinely ask questions about their patients’ sexual behaviour [10,11]. Furthermore, most MSMs do not disclose their sexual orientation to their GPs [11]. Nevertheless, most patients report a willingness to discuss their sexuality with their GPs [12]. These communication barriers result in missed opportunities for determining whether patients are at risk and thus eligible for screening. There is a growing consensus that GPs lack tools to assist them in reducing the population of undiagnosed people.

In this study, a questionnaire was developed specifically for use in general practice to identify risk factors for HIV, HBV and HCV aiming to facilitate joint screening. We evaluated two strategies for administering the questionnaire: a self-administered questionnaire, completed directly by patients, and a GP-administered questionnaire, in which GPs posed the questions orally while taking the patient’s medical history. We aimed to assess its acceptability regarding the overall response rate in general practice, and its effectiveness, in terms of its ability to identify patients most at risk and to increase the number of tests proposed. To provide guidance on administration, we compared the two administration strategies.

## Methods

### Study design

We performed an observational study in a prospectively enrolled cohort of patients from 18 general practices between March and July 2014.

We designed a questionnaire to detect risk factors of HIV, HBV and HCV, in line with both national and international guidelines [4–9]. The study’s steering committee validated the questionnaire.

### Participants

Two hundred GPs were randomly drawn from a list of private Paris-based general practices, based on a recent demographic study [13]. We contacted all 200 GPs and invited them to participate in the study. We asked GPs to provide their previous annual report of the French National Health Insurance scheme, which enabled us to collect the average number of consultations they had done during previous year and age range of their patient population. GPs were randomly assigned to two groups: A (self-administered) and B (GP-administered). A researcher who was not involved in the study performed randomization. The first 50 patients attending the participating GPs, irrespective of the reason for the consultation, were included if they were aged between 18 and 65 years old, came unaccompanied to the consultation and were able to speak and write French.

### Intervention

GPs provided enrolled patients with a self-administered questionnaire if they were in group A, or, if there were in group B, asked the same questions orally. The questions posed were the same for both groups and covered patients’ sexual histories (number and sex of sexual partners in the past 12 months), drug use, blood transfusions before 1992, diagnosed sexually transmitted infection (STI), tattoos or piercings and demographic information (see Supplementary material, available online). Based on the responses provided, the GPs used their discretion to determine whether to prescribe HIV, HBV and/or HCV tests to their patients, thus mimicking routine practice conditions to the greatest extent possible. For each patient questioned, GPs also indicated whether or not the patient was new and whether the risk factors identified via the questionnaire were documented in patient’s medical records or had been previously mentioned.

### Outcomes

We used the overall response rate to assess the acceptability of our questionnaire in general practice. Response rates between groups (A versus B) were then compared to determine whether one administration strategy was preferable to another.

We performed two analyses to assess the effectiveness of our questionnaire. We first estimated the proportion of risk factors newly identified during the study among patients already known by the GP (new patients excluded). To assess the number of tests carried out by their patients for each GP, we used data extracted from the French National Health Insurance database. We used the retrieved data to perform the second analysis, a pre–post comparison of the average number of HIV, HBV, and HCV tests carried out by patients per GP, over a period of four weeks, one year before our intervention compared to a period of four weeks immediately after the beginning of the study.

To assess the patients’ pathway from indication to realization of screening tests during the study, we studied patients with an indication for each test (i.e. who had a risk factor or who had never been tested before, according to the questionnaire). We calculated the proportion of patients who were offered each test by their GP, who had a prescription for each test (according to the doctor questionnaire), and who performed it in the laboratory (according to the health insurance database).

### Statistical analysis

To reach the number of subjects required (NSR), we hypothesized a difference in response rate between the two modes of administration (self-reported questionnaire versus GP-administered questionnaire). We assumed that the response rate in one group would be 65% and the other group 95%, corresponding to a 30% difference with an alpha level of 0.05 and a power of 0.9. As we randomized the patients in clusters, we had to readjust the NSR with an inflation factor, which depended on the number of patients in each cluster and the estimated intra-class correlation coefficient. There were 50 patients per GP and we estimated the intra-class correlation coefficient to be 0.23, based on a study with a similar design [14]. The NSR was 392 in each group, thus requiring eight GPs per group.

The response rates were analysed by calculating rates with standard deviations and confidence intervals. We used Student *t*-tests for comparison of response rates between groups, and for before-after comparison of the number of tests. Prism5 and R 3.1.1 were used.

### Protection of personal data

The GPs gave their consent to use of their prescription records. Collected patient data were anonymized. The French committee on data in health research (CCTIRS), the French data protection authority (CNIL) and an ethics committee (CPP Ile-de-France-4) approved the study.

## Results

### GPs and patients

**Figure 1.**
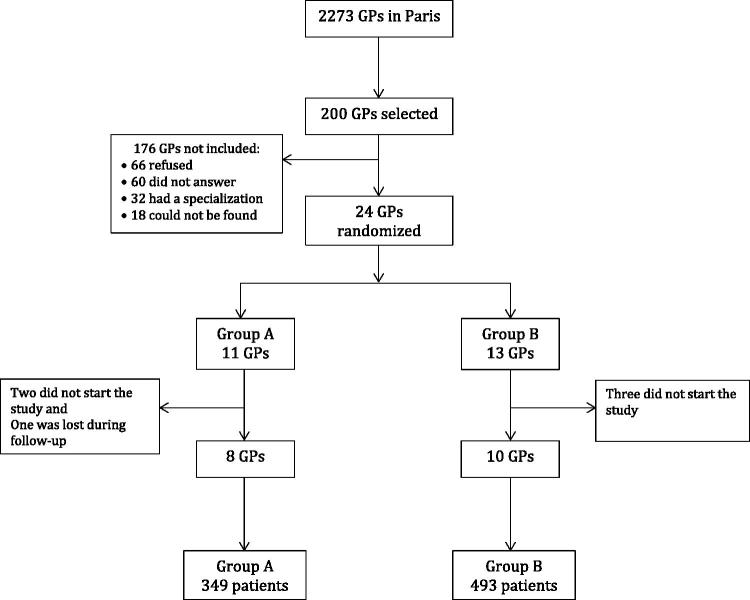
Flow chart.

Eighteen of the 200 GPs contacted ultimately participated in the study; eight were randomized to group A and 10 to group B. In our study, 842 patients were enrolled: 349 (41.5%) in group A and 493 (58.5%) in group B (Figure 1). Both groups were comparable concerning both the doctor and patient populations in our study. Fifty-five per cent of GPs included were women. Their average age was 53 years old. The average number of consultations per year per GP was 3829 and 76.9% of their patients were between 16 and 69 years old.

Included patients were between 18 and 65 years old and more women were enrolled. Most of the patients were born in France; those who were not from France were from North Africa, elsewhere in Europe or Sub-Saharan Africa (Table 1). There were 139/842 (16.5%) new patients (not known before the consultation).

**Table 1.** Demographic characteristics of included patients.

	Group A (*n* = 337)	Group B (*n* = 479)	*P*	All respondents (*n* = 816)
Age (years) average ± SD	36.6 ± 12.6	39.7 ± 13.2	0.82	39.7 ± 12.9
Gender F/M *n* (%)	221 (65.6)/116 (34.4)	271 (56.6)/208 (43.4)	0.01	492 (60.2)/234 (39.7)
Country of birth: France *n* (%)	249 (71.4%)	296 (60.0%)	0.13	545 (64.7%)

### Response rate to the questionnaire

Out of 842, 745 patients completed the questionnaire (response rate: 88.5%, with a 95% confidence interval [95%CI: 86.3–90.6]). The response rate in group A was higher than in group B, 93.1% was (95%CI: 90.5–95.8} versus 85.2% (95%CI: 82.1–88.3). The difference between groups was statistically significant (*P* = 0.0004).

### Prevalence of risk factors

Of the 745 patients who answered the questionnaire, 386 (51.8%; 95%CI: 48.2–54.4) had at least one risk factor for HIV, HBV or HCV (prevalence of risk factors: 51.8%; 95%CI: 48.2–55.4). The prevalence was 55.3% (95%CI: 49.5–61.1) in men and 49.3% (95%CI: 44.7–53.9) in women. The most common risk factors were tattoos/piercings, multiple partners in the last 12 months and having a prior STI diagnosis. Fourteen per cent of men in the study (95%CI: 10.3–18.5) were MSM. The prevalence of any risk factor did not differ between groups A and B (Table 2).

**Table 2.** Prevalence of risk factors among respondents to the questionnaires.

Risk factor (95%CI)	All respondents (*n* = 745)	Group A (*n* = 325)	Group B (*n* = 420)	*P*
Tattoos and/or piercings	23.0 (19.9–26.0)	23.3 (18.8–28.0)	22.6 (18.6–26.6)	0.79
Multiple partners in the last 12 months	17.1 (14.4–19.8)	20.0 (15.7–24.4)	14.8 (11.3–18.2)	0.06
History of sexually transmitted infections	15.2 (12.6–17.7)	13.9 (10.1–17.6)	16.2 (12.7–19.7)	0.41
Born in endemic country	11.8 (9.5–14.1)	9.2 (6.1–12.4)	13.8 (10.5–17.1)	0.07
Condoms not always used with a non-regular partner	9.8 (7.7–11.9)	11.1 (7.7–14.5)	8.8 (6.1–11.5)	0.36
Men having sex with men	5.5 (3.9–7.1)	4.3 (2.1–6.6)	6.4 (4.1–8.8)	0.26
History of blood transfusion before 1992	3.0 (1.7–4.2)	3.7 (1.6–5.7)	2.4 (0.9–3.8)	0.38
Intravenous drug use	2.0 (1.0–3.0)	1.8 (0.4–3.3)	2.1 (0.8–3.5)	0.61
High-risk population (at least one risk factor)	51.8 (48.2–55.4)	52.6 (47.2–58.0)	51.2 (46.4–56.0)	0.71
High-risk population ‘tattoos and piercings’ excluded	41.5 (37.9–45.0)	41.2 (35.9–46.6)	41.7 (37.0–46.4)	0.94

### Newly identified risk factors

Of the 386 patients with at least one risk factor, 321 were not new patients. Among them, GPs identified 187 patients with a risk factor they were unaware of before the consultation by using the questionnaire (58.3%, 95%CI: 52.9–63.7). Table 3 shows rates of newly identified risk factors for each risk factor. A greater proportion of risk factors were newly identified in group A compared to group B, 67.6% (95%CI: 59.8–75.5) versus 51.4% (95%CI: 44.1–58.6), respectively (*P* <0.05).

**Table 3.** Rates of newly identified risk factors, among patients with a risk factor, new patients excluded.

Risk factor (*n*)	Newly identified risk factors %
Tattoos and/or piercings (*n* = 134)	77.6
Multiple partners in the last 12 months (*n* = 103)	77.7
History of Sexually Transmitted Infections (*n* = 92)	70.7
Born in endemic country (*n* = 74)	70.3
Men having sex with men (*n* = 37)	10.8
History of blood transfusion before 1992 (*n* = 19)	68.4
Intravenous drug use (*n* = 13)	46.2
High-risk population: at least one risk factor (*n* = 321)	58.3

### Comparison of the number of tests before and after intervention

The average number of HIV tests performed by patients, per GP, over a four-week period, was 3.4 HIV tests (95%CI: 2.1–4.8) one year before the intervention. In the four weeks following the intervention, 4.4 HIV tests (95%CI: 3.3–5.5) were performed. The difference was not statistically significant (*P* = 0.22). However, HBV tests increased from 1.7 HBV tests (95%CI: 0.8–2.7) one year before to 3.7 HBV tests (95%CI: 2.4–4.9) after the intervention (*P* = 0.005). HCV tests increased from 1.4 HCV tests (95%CI: 0.7–2.2) before to 3.4 (95%CI: 2.1–4.7) after the intervention (*P* = 0.005).

### The screening cascade

**Figure 2.**
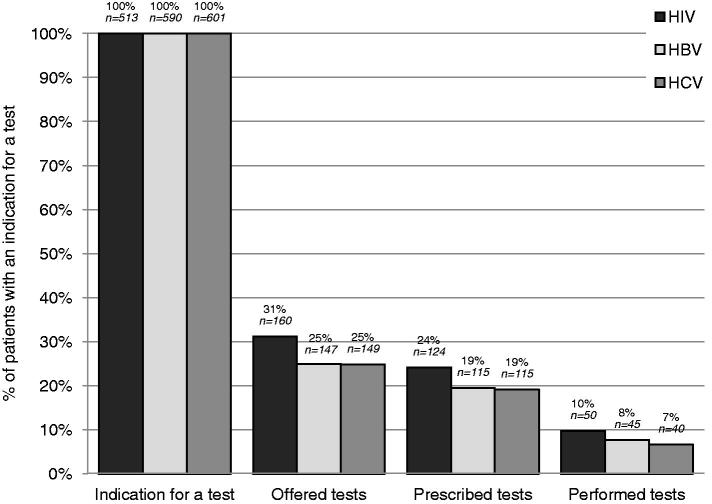
Screening cascade, from indication to realization of HIV, HBV and HCV tests. Percentages are calculated on the number of patients with an indication for a HIV, HBV, or HCV test (i.e. with a risk factor or who had never been tested before).

Depending on the virus, there was an indication for testing for 61–71% of patients included in the study. Among them, 25–31% were asked if they wanted to be tested for HIV/HBV/HCV, 19–24% received a prescription for a test at the end of the consultation, and 7–10% were ultimately performed at the laboratory (Figure 2).

## Discussion

### Main findings

The questionnaire developed for this study is a viable and effective tool for identifying risk factors for HIV, HBV and HCV in a general practice setting, independent of how it is administered. Higher responses rates in group A compared to group B suggest that patients may be more likely to complete a self-administered questionnaire rather than respond to questions posed orally by their GPs. The prevalence of risk factors was high, as more than half of patients had at least one risk factor. In most cases, GPs were unaware of the risk factor in question. In spite of the questionnaire’s ability to detect those at higher risk, GPs often failed to prescribe a test. Furthermore, among those who were prescribed a test, few participants carried it out.

### Strengths and limitations

Of the 200 GPs who were invited to participate in the study, only 18 ultimately participated (9%) (Figure 1). However, this figure does not differ from the proportion of French GP that participate in research, estimated at 10% in 2002 [15]. Still, we have to consider the possibility of a biased sample of GPs. As the proportion of those who participated in the study was low, those who agreed to participate could potentially represent those who are the most informed and interested. Nevertheless, our population of GPs was comparable to the population of GPs in Paris regarding their patient population and their age [16].

The study was conducted in 2014. GPs screening activity has not changed much in France since then, as it increased in 2015, compared to 2013, by only 3% for HIV [17], 6% for HBV and 8% for HCV [18]. The results of this study therefore are still of relevance.

There could have been a limited selection bias regarding the inclusion of patients by GPs. GPs were supposed to include the first 50 patients they saw without selecting them. Based on GPs previous annual report of the French National Health Insurance, we know that 76.9% of their patients were between 16 and 69 years old. We also know that in France, 66% of patients come unaccompanied to their GP [19]. From this data, we estimate that each GP should have seen in average 7.9 patients per day meeting the inclusion criteria. Each GP effectively included 3.7 patients per day, which represent 48% of the calculated number of patients, approximately one in two patients who came to them during the intervention.

Data on tests carried out in laboratories only include patients enrolled in the general health insurance scheme in Paris who were reimbursed for an HIV, HBV or HCV test, prescribed during the four weeks following the beginning of the study and performed within three months. Patients from other health insurance schemes (such as students or public sector employees) or from a health insurance scheme outside of Paris or without a health insurance were not included in this analysis. The period of four weeks after the consultation is quite conservative and may have led to an omission of some of the tests carried out. This may have resulted in an underestimation of the number of performed tests and makes it hard to conclude a learning effect.

### Newly identified risk factors

Many risk factors were identified during our study. The most common risk factor was to have tattoos and/or piercings (23.0%). While the role of tattoos and piercings for the transmission of HIV, HBV and HCV is hard to document, tattoos and piercings are still considered to be risk factors according to some guidelines [7–9]. Recent studies showed that the risk of infection for HBV and HCV did not increase in people with tattoos and/or piercings, compared to those without them as long as they were performed by a professional [20,21]. When we excluded tattoos and piercings of the risk factors, the risk population remained fairly high (41.5%) and the difference compared to the prevalence of all risk factors (51.8%) was not found to be significant. This is likely due to tattoos and piercings being associated with other risk factors.

In our study, GPs were not used to asking their patients questions to determine whether he/she was at risk for HIV, HBV or HCV (58.3% of the risk factors were newly identified). A 2013 study on missed opportunities for HIV screening in France reported that GPs did not discuss their patients’ sexual behaviour [22]. However, a study in Switzerland highlighted that patients are willing to talk about sexuality with their GP [12].

In 2009, only 1% of the GPs in France used a pre-established questionnaire with questions about sexuality [23] while it can help them to ask questions about sexuality to their patients.

### Prescription of screening tests

The gap between patients for whom a test should be offered and those who ultimately carried them out ended up being very wide. GPs were not selected and not trained before the intervention. In two French counties, selected GPs were asked to prescribe joint HIV, HBV and HCV tests routinely to patients who had never been tested before [24]. Screening tests were offered to 50% of the patients, prescribed to 38% of them and performed in laboratory for 14% of them. The rates of prescription were much higher in this study compared to our study, probably due to the recruitment of GPs who were already motivated and then trained during the study. Another study in London (UK) demonstrated that trained GPs performed more HIV tests after being trained on sexual health [25]. These results speak to the importance of providing clinicians with proper training aimed at improving screening practices. However, the lack of training is probably not the sole explanation for the low levels of tests carried out. Reluctance to go to the laboratory could be another explanation. Point-of-care testing with rapid tests for HIV, HBV, and HCV offered at GP’s office could be more acceptable and help to decrease the loss in follow-up after prescription of a test.

### Implications

The high response rate observed in both groups suggests that clinicians found our questionnaire acceptable. It could be used as a pre-established questionnaire, administered as part of recording new patients’ medical histories. GPs could integrate the essential questions of the questionnaire in the same way most of them do for cardiovascular or hereditary diseases. Our study suggests that our questionnaire can facilitate and assist GPs with more targeted screening for HIV, HBV, and HCV.

In France, in 2015, despite a slight increase in HIV testing activity, the number of confirmed positive tests remained stable [17]. This increase of testing is mainly due to guidance that recommended generalized screening for HIV, HBV, and HCV [8,9]. This example from France highlights the limitations of screening to reach populations most at risk of exposure to these infections. Screening is an essential step in the fight against STI. Diagnosing 90% of people living with HIV is the first aim of the Joint United Nations Programme on HIV/Acquired Immune Deficiency Syndrome (UNAIDS) 90-90-90 targets, which aim at ending HIV as a public health threat by 2030 [26]. The gap between our results and the 90% target underscores the need for more focused, strategic targeting of testing services to ensure 90% of those living with HIV are aware of their status, including high-risk populations. New tools are needed to improve targeted screening, in particular in GPs’ offices, and our questionnaire could be one of them.

The World Health Organization recently published guidelines about brief sexuality-related communication (BSC), which is defined as an opportunistic communication process in primary healthcare to address sexuality and related personal and psychological problems as well as to promote sexual wellbeing [27]. The use of our questionnaire could be the first step of this process, allowing primary care providers to personalize their communication with the patient.

## Conclusion

Our study shows that a questionnaire can help GPs identify risk factors for HIV, HBV, and HCV and improve screening practices for HBV and HCV. These results highlight the need to aid GPs in overcoming the taboo of talking about the sensitive subjects linked to the transmission of HIV and hepatitis and empowering them to effectively support patients in ‘knowing their status’. Our study also highlights that the list of risk factors in the current French recommendations is likely too broad and should potentially be more restrictive to screen more efficiently those at the highest risk.

## Supplementary Material

Supplemental material: questionnaire
