# Supplementary material for: The acceptability and effectiveness of a questionnaire for the identification of risk factors for HIV and hepatitis B and C: An observational study in general practice
Source: Eur J Gen Pract. 2017 Nov 27;24(1):60–7. doi: 10.1080/13814788.2017.1400529 (PMC5795630; doi:10.1080/13814788.2017.1400529)
Supplement: Supplemental material: questionnaire [file IGEN_A_1400529_SM4643.pdf]

Questions to ask all unaccompanied patients, aged 18 – 65 years old

Sex: *man/woman*
Age: __ years
Country of birth:

Lifestyle

Do you have any tattoos or piercings?	*Yes*	*No*
Have you had a blood transfusion prior to 1992?	*Yes*	*No*
Have you been drunk in the last 12 months?	*Yes*	*No*
Have you ever taken drugs?	*Yes*	*No*

If yes, which ones

Cannabis	*Poppers*	*Ecstasy*	*MDMA*
Amphetamines	*LSD*	*Crack*	*Cocaine*
Heroine	*GHB*	*Other drugs*	

If yes, have you ever injected drugs?	*Yes*	*No*

Have you ever had a sexually-transmitted infection or one transmitted by blood?
Yes *No*

If yes, which ones:

Gonorrhoea	*Chlamydia*	*Herpes*	*Syphilis*	*HPV*
HIV	*Hepatitis B*	*Hepatitis C*	*Other*	

Sex life

Do you currently have a regular sexual partner?		*Yes*		*No*
If yes, is this person a woman or a man?		*A woman*		*A man*
If yes, do you use condoms with this partner?	*Always*	*Often*	*Rarely*	*Never*

In the last 12 months, have you had other sexual partners?	*Yes, one*	*Yes, several*	*No*
If yes, were these persons…	*Women*	*Men*	*Both*

If yes, do you use condoms with these partners?	*Always*	*Often*	*Rarely*	*Never*

HIV, hepatitis, and you

Have you ever had sex with an HIV positive person?	*Yes*	*No*
Have you ever been tested for HIV?	*Yes*	*No*

If yes, when were you last tested?

> 6 months *6-12 months* *1-4 months* *5 years or more*

Have you ever been tested for hepatitis B?	*Yes*	*No*
Have you ever been tested for hepatitis C?	*Yes*	*No*
